# Grapevine disease detection using (q,τ)-nabla calculus quantum deformation with deep learning features

**DOI:** 10.1016/j.mex.2025.103619

**Published:** 2025-09-10

**Authors:** Ahmad Sami Al-Shamayleh, Rabha W. Ibrahim

**Affiliations:** a Department of Data Science and Artificial Intelligence, Faculty of Information Technology, Al-Ahliyya Amman University, Al-Salt, Amman 19328, Jordan; b Information and Communication Technology Research Group, Scientific Research Center, Al-Ayen University 64001, Thi Qar, Iraq

**Keywords:** Grapevine disease detection, Feature extraction, (q,τ)-Nabla calculus quantum deformation, Classification

## Abstract

Today, one of the most important first steps in attaining sustainable agriculture and guaranteeing food security is the detection of plant diseases. Quantitative analysis of plant physiology is now feasible thanks to developments in computer vision and imaging technologies. On the other hand, manual diagnosis requires a lot of work and in-depth plant pathology knowledge. Numerous innovative methods for identifying and classifying plant diseases have been widely used. In this study, we propose a novel hybrid classification method that combines (q,τ)-Nabla calculus quantum deformation-based features with deep learning feature representations to classify diseases in grapevine leaves. The methodology of this study relies on:

• Nabla calculus quantum deformation features are utilized to extract robust handcrafted features that capture local texture and structural variations associated with disease symptoms.
• Deep features are extracted using a pre-trained convolutional neural network, which captures high-level semantic information from leaf images.

The concatenated feature vectors are then fed into a machine learning classifier for final prediction. Test results on a dataset of grapevine leaf disease show that the proposed method outperforms individual approaches, in accuracy. The proposed method helps minimize financial losses and support effective plant disease management, thereby improving crop yield and contributing to food security.

## Graphical abstract

**Figure:**
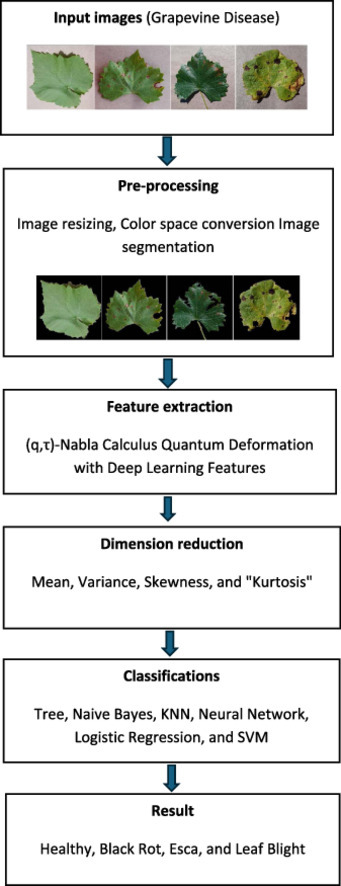

**Specifications table**

**Table:** 

**Subject area**	Computer Science
**More specific subject area**	Image processing
**Name of your method**	Grapevine disease detection from images
**Name and reference of original method**	Not applicable
**Resource availability**	https://www.kaggle.com/datasets/pushpalama/grape-disease

## Background

**Fig. 1:**
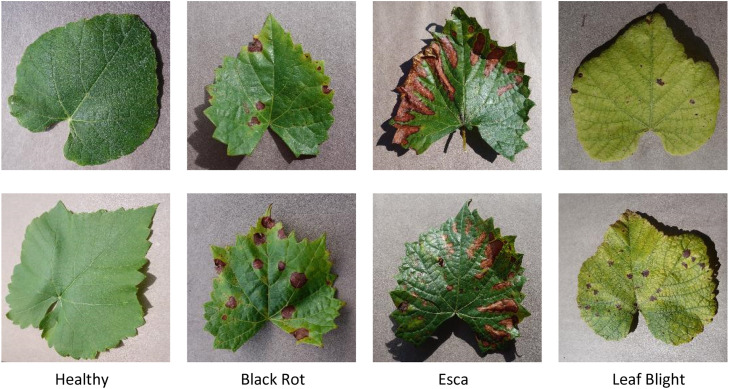
Sample of grapevine leaf diseases.

Grapevine cultivation is a significant part of Jordan’s agricultural landscape, especially in regions such as the Jordan Valley, Irbid, and Madaba, where favorable climate conditions support the growth of various grape varieties. Grapes serve as an essential crop not only for local consumption but also for dried products. However, the productivity and quality of grapevine yields in Jordan are increasingly threatened by the spread of leaf diseases. These diseases can severely reduce crop output if not detected and managed at an early stage. The three most prevalent grapevine leaf diseases are depicted in Table 1 and Fig. 1 [1,2]. Particularly in large-scale vineyards, traditional disease detection techniques that depend on expert manual inspection are frequently laborious, arbitrary, and error prone. To address these limitations, automated disease detection systems using image-based analysis and machine learning have emerged as powerful alternatives [3]. Identifying and classifying plant diseases poses a significant hurdle because of the vast diversity of plant species globally and the numerous ailments that hinder crop production [4]. In recent years, deep learning has shown remarkable performance in image classification tasks due to its ability to automatically extract high-level features from raw image data [5,6]. On the other hand, handcrafted features derived from mathematical models can provide meaningful complementary information, improving model robustness. This study proposes a machine learning-based approach for grapevine disease detection that combines (q,τ) NC-QD features with deep learning features. The (q,τ) NC-QD feature offers a novel mathematical technique for extracting texture and structural features from leaf images, which are often indicative of disease. These features are then combined with high-level deep features extracted using pretrained CNN, creating a powerful hybrid representation that captures both detailed texture and abstract visual patterns. The proposed method has the potential to be integrated into field-deployable platforms, making it practical for farmers to use directly in vineyards with minimal technical training. By leveraging both mathematical modeling and deep learning, the proposed system offers a promising solution for accurate disease detection in grapevines, enabling timely interventions and more sustainable vineyard management practices in Jordan.

**Table 1:** The common grapevine leaf diseases include causes, symptoms, and impact on the vine.

Disease	Cause	Key Leaf Symptom	Impact
Black Rot	Guignardia bidwellii	Small brown spots with black borders	Reduces fruit yield and quality. Severe infections can cause nearly complete crop loss if not controlled.
Esca	Fungal complex	Tiger stripes, interveinal yellowing or red	Can eventually kill the vine if the trunk becomes too damaged. Chronic disease — symptoms can disappear and reappear yearly.
Leaf Blight	Pseudocercospora vitis or others	Marginal necrosis, browning, and leaf drop	Reduces photosynthesis and vine vigor. If defoliation occurs early, fruit development may be severely affected.

## Method details

**Fig. 2:**
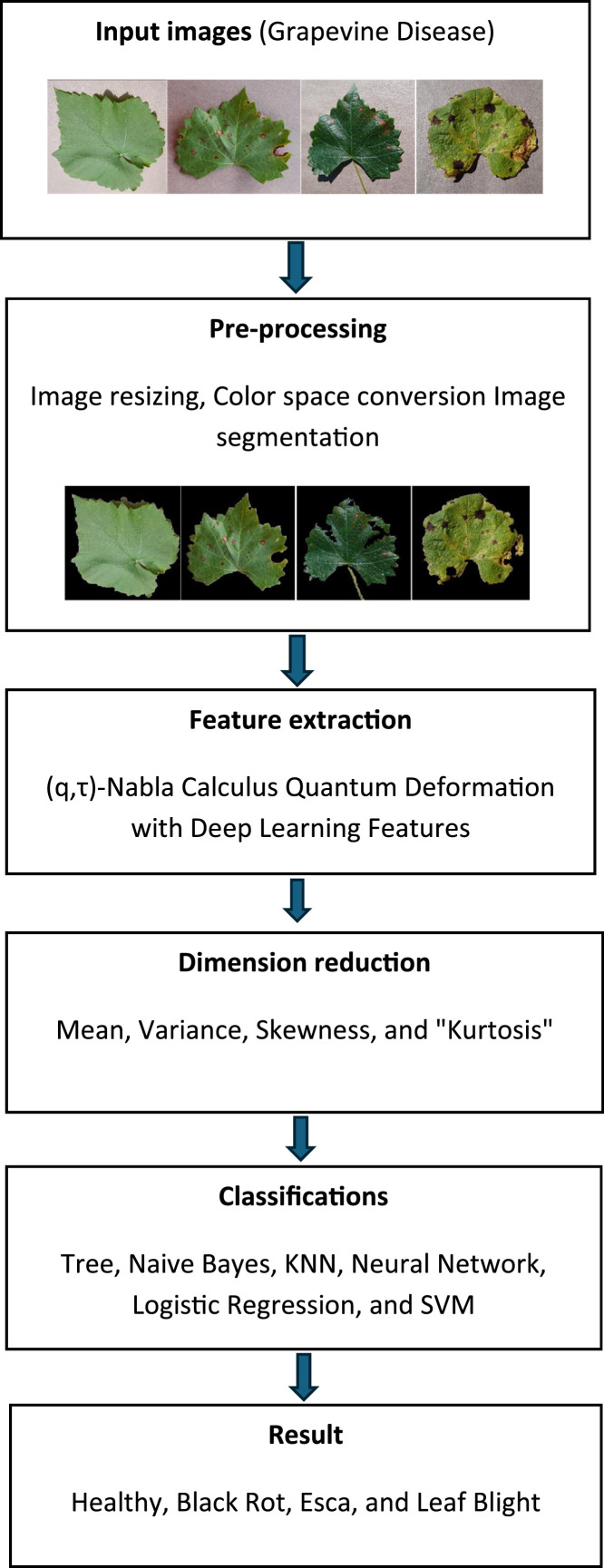
Block diagram illustrating the image preprocessing.

Plant disease detection for quantitative plant physiology analysis has become simpler thanks to developments in computer vision and imaging, which is a positive step toward sustainable agriculture and food security. Due to their economic significance, grapevine diseases are receiving more attention in recent research that has investigated a variety of machine learning approaches for plant disease detection. Traditionally, methods like Local Binary Patterns (LBP) [7], Histogram of Oriented Gradients (HOG), and Gray-Level Co-occurrence Matrix (GLCM) are used to extract handcrafted features like color, texture, and shape [3]. Although somewhat successful, these approaches have trouble handling the intricate differences in how diseases manifest. On the other hand, by automatically extracting features from images, Deep Learning (DL) models in particular, Convolutional Neural Networks (CNNs) performed better. Researchers have used architectures like AlexNet, VGG16, and ResNet to classify grapevine leaves with encouraging accuracy [8]. Nevertheless, CNNs frequently need a lot of data and a lot of processing power. Ferentinos et al., [8] (2018) proposed a DL approach for plant disease detection using CNNs architectures (AlexNet, GoogLeNet), including grapevine diseases. The model achieved up to 99.5 % accuracy on test sets. The significance of this study was to demonstrate the power of DL on plant pathology. However, some classes had many samples, while others had relatively few which can bias the model toward limiting its generalization to rare or early-stage infections. In their study,Amara et al [9] proposed disease classification method for grapevine and tomato. This study applied pretrained AlexNet and GoogleNet and achieved over 96 % accuracy on grape leaf disease detection. The significance of this study was that it reduces the need for large datasets. The main limit was no comparison with non-DL methods. In order to detect grape and mango leaves diseases, Sanath Rao et al [10] proposed transfer learning by repurposing a pretrained CNN (AlexNet) to extract features and categorize leaf images. The fine-tuned model achieved high accuracy on both crops. Most training images were captured in controlled conditions. In contrast, real-world conditions involve varying lighting, shadows, backgrounds, and overlapping leaves. GrapeGAN-based model to enhance grape leaf images to improve disease recognition was presented in the study by Jin et al, (2022) [11]. GrapeGAN preserves fine textures and structural integrity achieved classification accuracy to 96 % with VGG16. However, its evaluation is limited to augmented datasets, lacking validation on real-world field images. It also omits comparisons with other data‑augmentation methods and does not analyze computational costs or deployment feasibility on resource-constrained devices. Employing Grad-CAM visualization and aggressive data augmentation with EfficientNetV2L, Chandrasekar Venkatachalama et al, (2025) [12] introduced Advanced Grape Leaf Disease Diagnosis. The objective is to improve precision in grape leaf disease detection while enhancing model interpretability. However, the approach is tested only on curated datasets, omitting real‑world field images. It also lacks quantitative analyses of augmentation impact and computational resource profiling. The hybrid feature extraction approach, which combines handcrafted features and DL, provides a more thorough and reliable representation of the image features and improves performance across a range of classification tasks [13]. It has been noted that these hybrid approaches enhance classification performance, particularly when there is a class imbalance or little data [5]. In their study, Jain & Periyasamy et al., (2022) [14] proposed feature extraction method using a pretrained CNN with Random Forest classifier. This study achieved 91.66 % accuracy in disease classification. The study does not clearly establish why or how transfer learning outperforms traditional approaches in this context. By leveraging of parallel CNN-based feature extractors (VGG16, InceptionV3, Xception, ResNet50) followed by a Random Forest classifier, Ishengoma et al., (2024) [15] proposed an ensemble method for grape leaf disease detection. This approach achieved up to 95.3 % accuracy. However, its limitations include dependence on handcrafted preprocessing, lack of evaluation under real-world vineyard conditions. Moreover, Rasika G. et al. (2024) [16] presented a system that uses fused DL features to diagnose grape leaf disease. They extract deep features from three CNN models across multiple layers, fuse them, and classify diseases via a multi-class SVM. They achieved accuracy of 99.58 %. However, the study relies on a single curated dataset, lacking validation on field-acquired images. The study by Sherihan Aboelenin et al., (2025) [4] proposed a hybrid DL framework combining CNNs and a Vision Transformer (ViT) to improve plant leaf disease detection and classification. This model achieved outstanding accuracy (99 % for Apple and 98 % for corn). However, the model has been evaluated on curated datasets, raising concerns about generalization to diverse real-world conditions. In order to detect the grape leaf diseases, Wu Canghai et al, (2025) [17] proposed a fine-grained recognition system by combining transfer learning with a Convolutional Block Attention Module (CBAM) appended to a ResNet50 backbone. The method achieved accuracy 97 %. However, its evaluation remains confined to curated, lab-quality images with a limited number of classes. While manual feature extraction created simpler models with less processing overhead, deep learning models often require a lot of processing power. DL and hand-crafted features have been used in hybrid approaches that appear to close these gaps. By retaining semantic knowledge from deep networks while capturing fine-grained texture patterns, these methods take advantage of the complementary benefits of both feature types. The primary contribution of this research is the creation of a feature extraction model based on a DL model with (q,τ) NC-QD features for classifying grapevine leaf diseases. Feature extraction plays a key role in the classification of grapevine leaf diseases by enabling the transformation of leaf images into meaningful and unique representations. Data collection, pre-processing, feature extraction, dimension reduction, and classification are all included in the proposed study as illustrated in Fig. 2.

### Image dataset

**Fig. 3:**
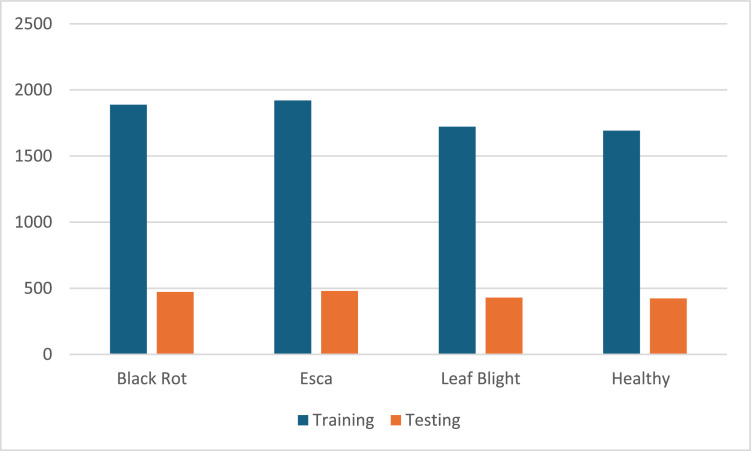
The four classes of image dataset.

The grapevine disease dataset was gathered from kaggle.com [18]. Fig. 3 shows the four classes of images used: Esca, black rot, leaf blight, and healthy. A total of 7222 images were used for training, and 1805 images were used for testing.

### Image preprocessing

Pre-processing usually involves converting the data into a format better suited to the classification algorithm. In this study the pre-processing of the image includes the following stapes.

### Image resizing

Resizing the images to 224 × 224 pixels as part of the data preprocessing process made it possible to use a range of transfer learning models during the testing phases.

### Color space conversion

The grapevine leaf diseases often appear as discoloration in images, so that it is necessary to preserve color information in the grapevine leaf making the classification process more efficient. The RGB color space, which is composed of the red, blue, and green components, was changed from the original images to the YCbCr color space. The YCbCr color space can be a good option for leaf classification tasks because it can distinguish between components of chrominance (Cb and Cr) and luminance (Y). Regardless of lighting conditions, the YCbCr color space separation facilitates the focus on color information (Cb and Cr) for color-based classification tasks.

### Image segmentation

**Fig. 4:**
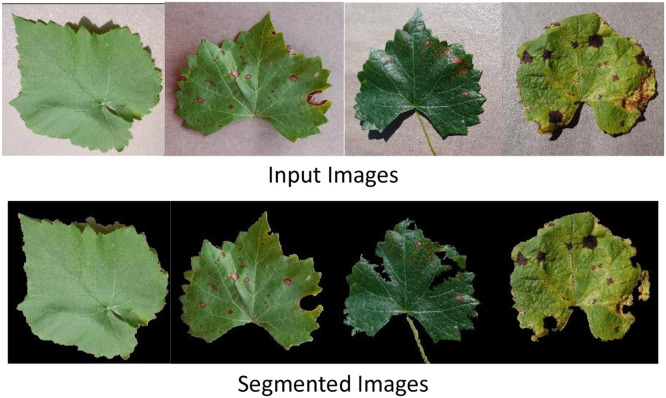
Sample of segmented grapevine leaf diseases mages.

The images of grapevine leaf diseases are segmented during image preprocessing in order to remove the leaf portion by suppressing the background pixels. The chrominance and luminance colors in the image are used to determine these threshold values. Fig. 4 displays the results of the image segmentation step. The classification of grapevine leaf diseases can be greatly improved by using the YCbCr color space and background segmentation. In order to guarantee that the suggested feature extraction models concentrate on the features that are most important, grapevine leaf diseases separated the background from the extraneous portions of the image.

### Proposed features extraction

The aim of the feature extraction algorithm is to take the preprocessed images of grapevine disease and extract the feature set that is most useful and helpful. These features include (q,τ)-Nabla calculus in quantum deformation (q,τ) NC-QD and DL features.

### Proposed (q,τ) NC-QD feature extraction

A novel (q,τ)-fractional difference operator by utilizing the generalized (q,τ)-Gamma function is introduced in this study. This operator extends the classical fractional difference in discrete calculus and is shown to preserve linearity and consistency with the standard difference operator in the limit q→1, τ→1. A formal proposition is established, and its proof is provided in detail.

Let 0<q<1, τ>0, and define the (q,τ)-Gamma function as:

(1) $$\Gamma_{q,\tau }\left(x\right)≔{\left(1-q\right)}^{1-x}\prod_{k=0}^{\infty }\frac{1-q^{\tau \left(k+1\right)}}{1-q^{\tau x+\tau k}}.$$

This generalizes the classical Gamma function and reduces to it as q→1 and τ→1.

Define the fractional (q,τ)-nabla operator for discrete functions x(n) as:

(2) $$\;\nabla_{q,\tau }^{\alpha }\;x\left(n\right)≔\frac{1}{\Gamma_{q,\tau }\left(1-\alpha \right)}\sum_{k=0}^{n-1}\frac{x\left(k\right)-x\left(k+1\right)}{{\left(n-k\right)}_{\left(q,\tau \right)}},$$

where ${\left(n\right)}_{q,\tau }=\;\frac{1-q^{\tau n}}{1-q^{\tau }}$ is a suitable normalization depending on the (q,τ)-difference scale, $\nabla_{q,\tau }x\left(n\right)\;$is the (q,τ)-difference element and$\;x_{k}$ are nodes (or any suitable (q,τ)-grid) .

(3) $$\;\nabla_{q,\tau }^{\alpha }\;x\left(n\right)≔\frac{1}{{\left(1-q\right)}^{\alpha }\prod_{k=0}^{\infty }\frac{1-q^{\tau \left(k+1\right)}}{1-q^{\tau \left(1-\alpha \right)+\tau k}}}\sum_{k=0}^{n-1}\frac{x\left(k\right)-x\left(k+1\right)}{\left(\frac{1-q^{\tau \left(n-k\right)}}{1-q^{\tau }}\right)}$$

where x is the value of an image pixel and α is the fractional power. In the experiment, α was set to equal 0.8, q (quantum number) to 0.5, and τ (scaling) to 1.2. These selected criteria were used for ensuring stability, capturing color shifts in grapevine leaves, and providing balanced, distortion-free discriminative features. The benefit of employing quantum-inspired operators for feature extraction is their capacity to extract texture and edge features from images, with the sensitivity of intensity changes and local variations being controlled by their parameters (q,τ).

**Fig. 5:**
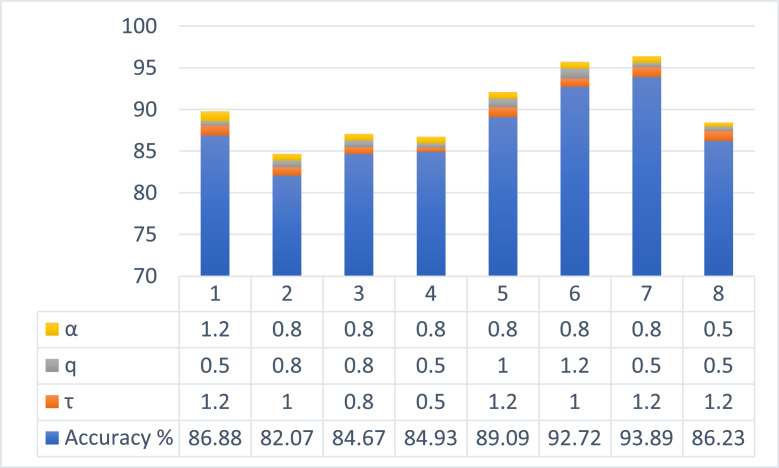
The performance of proposed (q,τ) NC-QD feature extraction by varying the scaling parameter (τ), the quantum number (*q*), and the fractional power(α).

The performance of proposed handcrafted (q,τ) NC-QD feature extraction on grapevine disease detection was experimentally evaluated by varying the scaling parameter (τ), the quantum number (*q*), and the fractional power(α). Fig. 5 showed how well the suggested (q,τ) NC-QD feature extraction performed when the parameters τ, *q*, and α were changed. According to the results, there are clear trends showing that the interplay of these three parameters affects classification accuracy.The parameter that had the biggest impact on detection accuracy was the scaling parameter τ. Improved classification performance was consistently associated with higher values of τ (1.2), with a maximum of 93.89 %. Lower values (τ=0.5), on the other hand, decreased accuracy to the 84–85 % range. This implies that the discriminability of textural features associated with disease is improved by greater scaling parameters. As a fine-tuning factor, the quantum number *q* modulated accuracy based on how it interacted with τ. This suggests that under ideal deformation conditions, discriminative information is amplified by lower *q* values. There was a stabilizing impact from the fractional power α. The best overall accuracy (93.89 %) and consistently better results were obtained with α=08. The sensitivity analysis shows that whereas α mainly stabilizes the extraction process, accuracy is jointly dictated by the interaction between τ and *q*. With an accuracy of 93.89 %, the ideal configuration was found to be (α=0.8, *q* = 0.5 and τ=1.2).

Dividing the images into non-overlapping blocks of 16 by 16 pixels is the first step in the feature extraction process. A simple reflection padding approach has been employed to manage boundaries, providing that every image is completely tiled by 16 × 16 blocks without causing unnatural discontinuities at the margins. The dimension reduction of features is applied after each image's features have been extracted using Eq. (3). This ensures that the algorithm operates in the most efficient and successful configurations possible. The dimensions of the extracted data in each image are reduced in this study using the metrics of "Mean," "Variance," "Skewness," and "Kurtosis" [19]. These four instances were selected to minimize dimensionality and overfitting. Algorithm 1 provides an illustration of NC-QD feature extraction pseudocode.

**Algorithm 1:** 

BEGIN
INPUT image
// Step 1: Divide image into non-overlapping 16 × 16 blocks
blocks ← image, block_size = 16
// Step 2: For each block, compute features using Eq. (3)
FOR each block IN blocks DO
features ← [F]
feature_value ← apply_Eq3
END FOR
// Step 3: Apply dimension reduction metrics
mean_val ← compute_mean(features)
variance_val ← compute_variance(features)
skewness_val ← compute_skewness(features)
kurtosis_val ← compute_kurtosis(features)
// Step 4: Store reduced feature vector for this image
reduced_features.append( [mean_val, variance_val, skewness_val, kurtosis_val])
END FOR
// Step 5: Output final feature set for all images
OUTPUT final_feature_set
END

**Fig. 6:**
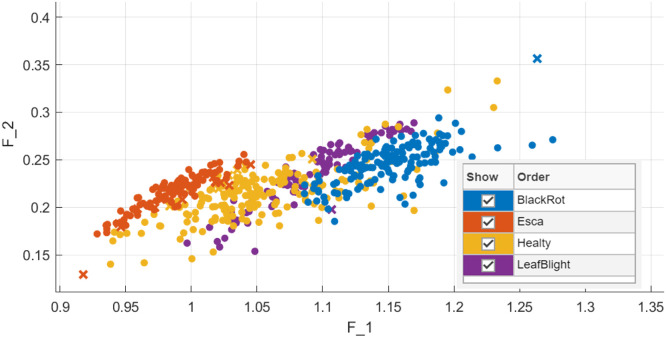
The four classes' features distribution Proposed (q,τ) NC-QD feature extraction.

The feature distribution is presented in Fig. 6 as a quantitative basis for feature separation.

### Pre-trained CNN (VGG16)

**Fig. 7:**
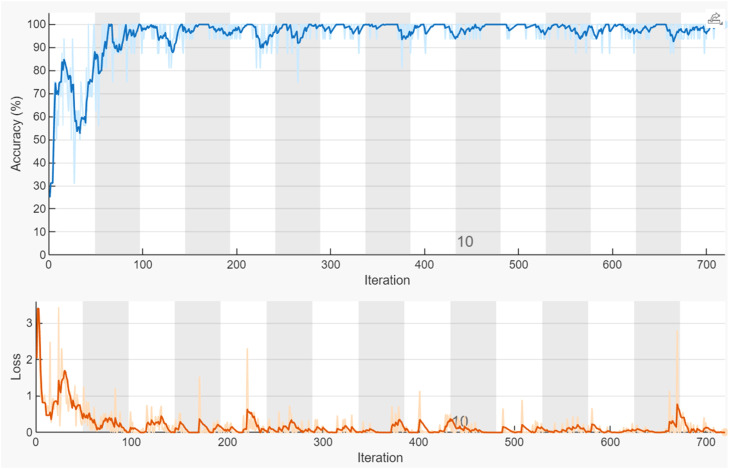
The training procedure for Pre-trained CNN (VGG16).

For image feature extraction tasks, using pre-trained CNNs rather than specially created CNNs to extract features from images has grown in popularity and effectiveness. To learn rich and generalizable feature representations, pre-trained CNNs like VGG16, ResNet, or Inception are trained on extensive datasets like ImageNet. These networks can capture hierarchical features that can be applied to a variety of visual tasks, such as edges, textures, and intricate shapes. The lower training time and computational cost of pre-trained CNNs is a significant benefit. Users don't need to start from scratch when extracting features because the network has already learned useful features. Since custom CNNs frequently need sizable, annotated datasets to achieve competitive performance, this is especially helpful when working with sparse data. Additionally, pre-trained models have been thoroughly examined and refined, guaranteeing a consistent baseline with high precision. On the other hand, creating a custom CNN necessitates extensive experimentation to identify the best configurations and knowledge of network architecture. Furthermore, when data is limited, training a custom model from scratch may result in overfitting. Consequently, employing pre-trained CNNs enhances robustness and generalization in feature extraction for image-based tasks while also accelerating development. The VGG16 Net "Visual Geometry Group" input layer has dimensions of 224 × 224 × 3. A 2 × 2 pool with five maximum pool layers. There are three primary sections in VGG16. Early and mid-level convolutional layers capture texture, form, and edge details at a low to mid-level. High-level semantic information is captured by fully connected layers, and Softmax/classification layers are not utilized for feature extraction, but rather for categorization. Training mode: Hardware resource: single GPU, Stochastic Gradient Descent, momentum = 0.9, Max epochs = 15 and learning rate = 0.0001 are the ideal hyperparameters that were found for training of trained CNNs - VGG16Net models. The VGG16 has been re-trained using datasets of images of grapevine diseases dataset, while the last three layers (fullyConnectedLayer, softmaxLayer, and classificationLayer) have been adjusted for 4-class classification. Fig. 7 shows the training procedure for refined CNN "VGG16" models along with the number of iterations.

## Method validation

In this study, the test was carried out using MATLAB 2025a utilizing an Intel Core i7–6700HQ CPU (2.6 GHz), 16 GB of RAM, 64-bit Windows 11, and an NVIDIA GTX 950 GPU. The performance of the machine learning algorithm is assessed by using nested 5-fold cross-validation, which splits the data into 5 equal-sized folds, 4 for training and 1 for testing. Each of the five folds in this process is tested once, and the remaining four folds are utilized for training. The results show that the performance of proposed grapevine disease detection was significantly improved by combining the extracted features. Fine Tree (FT), Kernel Naive Bayes (KNB), Fine KNN (FKNN), Narrow Neural Network (NNN), Logistic Regression (LR), and Quadratic SVM are the classifiers used in the proposed method. Table 2 displays the achieved accuracy with the kernel function for each classifier. The results show that SVM’s ability to handle high-dimensional and potentially non-linear feature spaces is demonstrated by its highest accuracy of 98.79 %. This implies that SVM is especially useful for differentiating between minute patterns in diseases of grapevine leaves. Naive Bayes and Logistic Regression demonstrated strong performance, achieving 96.52 % and 96.35 % accuracy, respectively. While Logistic Regression shows that a linear decision boundary can successfully divide most of the classes, Naive Bayes's strong performance suggests that the features may have some degree of conditional independence. The accuracy of the Neural Network classifier was 94.91 %, which is marginally lower than that of SVM and Naive Bayes, despite being high. This could be because of inadequate training epochs, suboptimal architecture, or a lack of training data. The accuracies of K-Nearest Neighbors (KNN) and Decision Tree were 91.18 % and 92.11 %, respectively, lower. While KNN can have trouble with high-dimensional data and is sensitive to the number of neighbors and distance metric selection, trees are prone to overfitting, particularly on small or noisy datasets.

**Table 2:** The classification results using different classifiers on grapevine disease dataset.

Classifiers	Accuracy (100 %)	Kernel function
Tree	92.11	Fine Tree
Naive Bayes	96.52	Kernel Naive Bayes
KNN	91.18	Fine KNN
Neural Network	94.91	Narrow Neural Network
Logistic Regression	96.35	Logistic Regression Kernel
SVM	98.79	Quadratic SVM

**Fig. 8:**
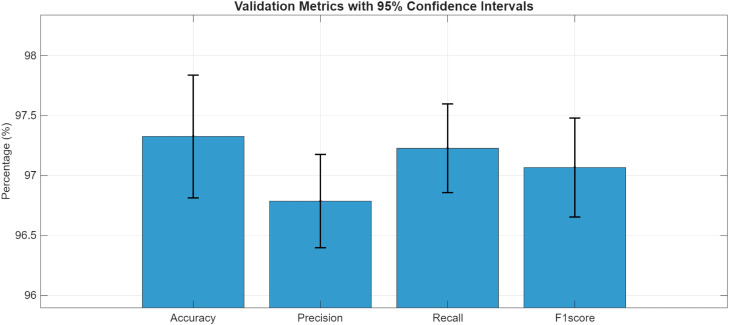
The classification results with 95 % Cis.

The suggested model demonstrated consistent classification performance throughout validation folds with an average accuracy of 98.79 % (SD = 0.55) and a 95 % confidence interval of [98.49 %, 99.10 %] as illustrated in Table 3. These findings demonstrate that the suggested method is appropriate for grapevine disease detection in a variety of settings. The mean classification performance over 5-fold cross-validation with 95 % CIs is displayed in Fig. 8. The grapevine disease detection method is highly effective, as evidenced by all metrics over 96 %. Low variability between folds is demonstrated by the narrow confidence intervals (< ±0.5 %), indicating the stability and robustness of the suggested model. Precision and recall are closely followed by the F1-score, which emphasizes the model's balanced performance in identifying all disease classes with negligible bias towards false positives or false negatives.

**Table 3:** The classification results of proposed model for grapevine disease detection.

Metric	Mean Accuracy (100 %)	Standard Deviation (SD)	Confidence Intervals Lower	Confidence Intervals Upper
Accuracy	98.79	0.5507	98.48	99.09
Precision	97.38	0.5044	97.10	97.66
Recall	97.56	0.4095	97.33	97.78
F1score	97.53	0.4205	97.3	97.76

**Fig. 9:**
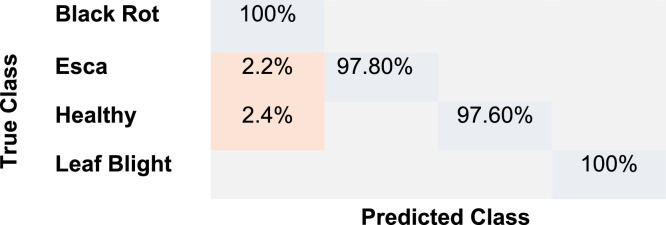
Confusion matrix for grapevine disease classification.

The confusion matrix in Fig. 9 shows how well the classification model performs in differentiating between the four grapevine leaf conditions—Leaf Blight, Black Rot, Esca, and Healthy. As demonstrated by the diagonal values near or precisely at 100 %, the model generally demonstrates high classification accuracy. There's some confusion between Esca and Black Rot (around 2.2 % misclassification) IN WHICH 2.2 % were incorrectly identified as Black Rot. Esca and Black Rot leaves may have similar texture patterns or overlapping color qualities, which could explain this little misunderstanding. The model exhibits good predictive power with little class confusion. There is potential for improvement, though, perhaps with more training data and better preprocessing, as indicated by the small misclassifications (most notably between Healthy and Black Rot).

The combination of deep learning, fractional quantum operator, and (q,τ)-nabla calculus yields an efficient benefit in grapevine disease diagnosis by capturing both fine-grained texture cues and high-level semantic context. Additionally, recollection was improved, especially for early symptoms and unusual disorders. All things considered, this hybrid strategy is ideal for practical use in vineyard monitoring systems, particularly on edge devices where dependability and effectiveness are crucial.

**Fig. 10:**
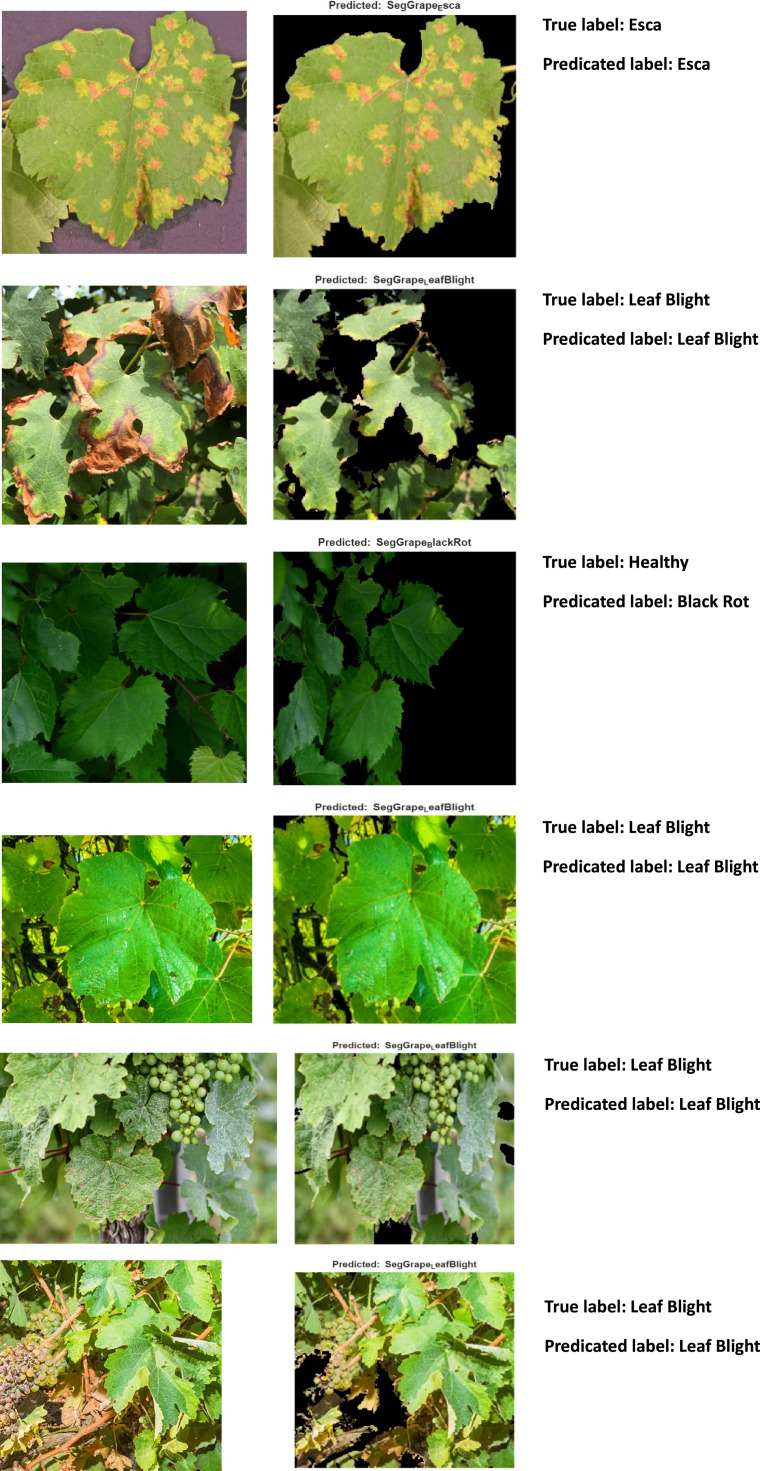
Sample of real-world images.

To expand assessment beyond reference databases to actual field images A sample of these photographs along with the prediction results is shown in Fig. 10. These sample images have many leaves, shadows, and ambient noise, which makes detection more difficult but more realistic than curated datasets (where leaves are chopped and isolated). The suggested approach uses segmentation, which isolates the leaf/disease areas and eliminates the backdrop. Segmentation is used in the proposed method to isolate the leaf/disease areas and remove the background. But some partial leaf occlusions and lighting changes (like the shadows in the third and fourth situations) might still make accuracy difficult. There are significant intensity differences between bright sunshine and shaded foliage. The classifier may have trouble generalizing, but it still produces illness classes. There were several overlapping leaves in the example of black rot. For prediction, segmentation had to separate the appropriate leaf. The last row displays a leaf that is largely healthy, yet the algorithm identified it as having "Leaf Blight," suggesting the possibility of false positives in specific lighting conditions or textural aberrations.

The illustration presented solid proof that the proposed method is practical in actual vineyard environments. While classification networks can differentiate between diseases such as Esca, Leaf Blight, and Black Rot, segmentation-based techniques aid in removing background clutter.

### Computational efficiency analysis

The computational efficiency of the proposed grapevine disease classification method was evaluated in terms of inference latency, throughput, and training time per epoch of the deep learning features as summarized in Table 4. The model demonstrated its capacity to produce predictions in close to real-time by achieving an inference latency of 5.396 ms per image. Diagnostic outputs can be produced nearly instantly thanks to this low response time.

**Table 4:** The computational efficiency analysis.

Inference latency	5.396 ms/image
Throughput	*185.3 images/sec*
Training time per epoch	*8.42* s

With a throughput of 185.3 pictures per second, the system showed excellent scalability for extensive vineyard evaluations. This performance level suggests that the proposed method can effectively manage scenarios involving batch processing, allowing for the quick screening of leaf samples during field inspections.

The model's computationally lightweight design was demonstrated by the 8.42-second training time per epoch. The proposed method is appropriate for iterative testing and adaptation to new datasets because of its brief training period, which also lowers the overall time and expense of model optimization.

The efficiency study, taken as a whole, demonstrates that the developed method is quick and resource-efficient, which increases its applicability for practical implementation. Low inference latency, high throughput, and low training overhead all work together to facilitate its use in grape disease monitoring in real time.

### Ablation study

In an ablation study, we investigated two studies. Ablation study 1 was conducted through three experiments that included altering the fc of VGG16 in which the features were extracted to assess their impact on the performance of feature extraction of the deep learning features. The ablation study's findings in Table 5 show that the classification accuracy of proposed grapevine disease detection significantly impacted by the feature extraction layer selection of VGG16. The final convolutional block's features (conv5_3) had an accuracy of 67.75 %, showing that robust illness classification requires more than just low- and mid-level spatial data. A notable performance increase of 95.05 % was obtained by extracting from the fc6, demonstrating the advantages of globally aggregated, high-level representations. The fully connected layer fc7 produced the maximum accuracy of 98.79 %, indicating that it offers the most class-specific and discriminative features.

**Table 5:** Ablation study 1.

Extracted features from fully connected layers (fc)	Test Accuracy ( %)
conv5_3	67.75
fc6	95.05
fc7	98.79

The ablation study 2, which is shown in Table 6, assesses how various preprocessing techniques affect classification performance. An accuracy of 89.14 % was obtained using the RGB color space, demonstrating the limits of direct RGB representation in managing background noise and light changes. By separating luminance from chrominance components, the YCbCr color space replaced RGB and increased accuracy to 95.82 %. This improves robustness to lighting conditions and highlights color-based disease patterns. Accuracy increased to 98.79 % by adding background segmentation. This improvement is probably the result of the model concentrating on disease-relevant leaf areas by eliminating unnecessary background regions. The findings show that background segmentation and color space transformation are both essential preprocessing processes, resulting in a total accuracy gain of 9.65 % over the baseline.

**Table 6:** Ablation study 2.

Preprocessing algorithms	Test Accuracy ( %)
RGB color space	89.14
YCbCr color space	95.82
Background segmentation	98.79

### Comparison with existing research

The performance comparison between the suggested feature extraction method and previous research using the same image dataset (grapevine disease dataset) as illustrated in Table 7. Rasika G. et al [16] used an ensemble of ResNet101 and ResNet50 to obtain the highest reported accuracy of 99.58 %. Although this is quite accurate, the usage of two deep architectures results in a significant computational expense. With the GC-MobileNet model, which is intended for mobile deployment, Wu Canghai et al [17] achieved 98.63 % accuracy, demonstrating its applicability in resource-constrained contexts. According to Chandrasekar Venkatachalama et al [12], EfficientNetV2L achieved 98.39 %, indicating remarkable performance despite a comparatively heavy backbone network. Prior studies that used deep features in conjunction with Random Forest classifiers, like Bhavya Jain et al [15]and Farian Ishengoma et al [15], reported lower accuracies (91.66 % and 95.34 %, respectively), indicating that feature extraction method selection and classifier integration have a substantial impact on performance. With an accuracy of 98.79 %, the proposed method outperformed EfficientNetV2L, GC-MobileNet, and Random Forest-based methods and came close to the ResNet ensemble's performance while providing a more computationally efficient solution. By combining Nabla calculus quantum deformation features with VGG16 deep features, this competitive precision is attained, allowing for a rich representation of both high-level semantic information and local texture patterns. The findings show that the suggested approach offers a good balance between classification accuracy and computational expense, which makes it a viable option for practical uses like in-field grapevine disease diagnosis. This efficiency is attained by eliminating the requirement for extremely deep or resource-intensive architectures by integrating compact quantum deformation features with lightweight CNN-based deep features. As a result, the proposed method is useful for field-based real-time sickness diagnosis with modest processing resources and maintains excellent accuracy on properly chosen datasets.

**Table 7:** Comparison with existing state-of-the art on grapevine disease dataset.

Methods	Technique	Accuracy %	Precision %	Recall %	F1-Score %
Chandrasekar V. et al, (2025) [12]	EfficientNetV2L	98.39	96.67	96.77	96.69
Bhavya Jain et al., (2022) [14]	VGG16 as a feature extractor and random forest as a classifier	91.66	91.75	91.50	91.50
Farian Ishengoma et al., (2024) [15]	CNN feature extractors and random forest classifier	95.34	95.20	95.25	95.5
Rasika G. et al. (2024) [16]	ResNet101 + ResNet50	99.58	99.67	99.68	99.68
Wu Canghai et al, (2025) [17]	GC-MobileNet model	98.63	98.57	98.59	98.57
Proposed method	Hybrid features: quantum deformation with Pre-trained CNN (VGG16) features	98.79	98.72	98.23	98.07

Grapes are a necessary crop for both local consumption and the production of dried goods. However, the growth of leaf diseases is posing a growing threat to grapevine yields' productivity and quality. If these diseases are not identified and controlled early on, they can significantly lower crop yield. This study proposed a novel hybrid classification method for identifying and categorizing diseases in grapevine leaves by fusing DL feature representations with Nabla calculus quantum deformation features. The robustness and classification accuracy of grapevine disease detection were enhanced by this new hybrid feature extraction method. Experiments were conducted using the publicly available grapevine Disease dataset. Comparative tests revealed that the SVM had the highest classification accuracy of all the classifiers, with an overall classification accuracy of 98.79 %. To improve the integration of DL features with Nabla calculus quantum deformation characteristics, future research directions for this study could involve investigating more complex feature fusion approaches. Additionally, the methodology may be applicable to different crops as well as, operational field trials are still required for deployment as part of future development.”

## Limitations

The focus of this study was to a novel hybrid classification method for identifying and categorizing diseases in grapevine leaves by fusing DL feature representations with Nabla calculus quantum deformation features. This study has some limitations regarding dataset dependency, such as datasets from different geographical locations and variations in imaging conditions, lighting, or surroundings. As well as considering real-world deployment.

## Ethics statements

This article does not contain any studies with human or animal participants.

## CRediT author statement

**Ahmad Sami Al-Shamayleh**: Methodology, Material preparation, Software; Validity tests. **Rabha W. Ibrahim:** Writing- Original draft, data collection and analysis.

## Declaration of competing interest

The authors declare that they have no known competing financial interests or personal relationships that could have appeared to influence the work reported in this paper.

## Data Availability

No data was used for the research described in the article.
